# Phylogeny of *Amazona barbadensis* and the Yellow-Headed Amazon Complex (Aves: Psittacidae): A New Look at South American Parrot Evolution

**DOI:** 10.1371/journal.pone.0097228

**Published:** 2014-05-13

**Authors:** Adam Dawid Urantówka, Paweł Mackiewicz, Tomasz Strzała

**Affiliations:** 1 Department of Genetics, Faculty of Biology, Wrocław University of Environmental and Life Sciences, Wrocław, Poland; 2 Department of Genomics, Faculty of Biotechnology, Wrocław University, Wrocław, Poland; University of Illinois at Urbana-Champaign, United States of America

## Abstract

The Yellow-shouldered Amazon (*Amazona barbadensis*) is the sole parrot of the genus *Amazona* that inhabits only dry forests. Its population has been dropping; therefore it has been the topic of many studies and conservation efforts. However, the phylogenetic relationship of this species to potential relatives classified within the Yellow-Headed Amazon (YHA) complex are still not clear. Therefore, we used more extensive data sets, including the newly sequenced mitochondrial genome of *A. barbadensis*, to conduct phylogenetic analyses. Various combinations of genes and many phylogenetic approaches showed that *A. barbadensis* clustered significantly with *A. ochrocephala ochrocephala* from Colombia and Venezuela, which created the Northern South American (NSA) lineage, clearly separated from two other lineages within the YHA complex, the Central (CA) and South American (SA). Tree topology tests and exclusion of rapidly evolving sites provided support for a NSA+SA grouping. We propose an evolutionary scenario for the YHA complex and its colonization of the American mainland. The NSA lineage likely represents the most ancestral lineage, which derived from Lesser Antillean Amazons and colonized the northern coast of Venezuela about a million years ago. Then, Central America was colonized through the Isthmus of Panama, which led to the emergence of the CA lineage. The southward expansion to South America and the origin of the SA lineage happened almost simultaneously. However, more intensive or prolonged gene flow or migrations have led to much weaker geographic differentiation of genetic markers in the SA than in the CA lineage.

## Introduction

According to the most recent classification of New World parrots, ten genera are ascribed to the Androglossini tribe [1]. One of them is the genus *Amazona* (Amazon parrots), which is represented by the largest number of species. It is widely distributed in Central and South America (from Mexico to Argentina), and throughout the Caribbean [2]–[4]. Amazon parrots are phenotypically characterized by their medium to large size, strong-heavy bill, short-rounded tail, prominent naked cere and the presence of a distinct notch in the upper mandible [3]. Their body plumage is predominantly green (except for a few Lesser Antillean species) with variable colorations on the head, breast, wing coverts, and/or flight feathers. The variation of these accenting colors is one of the morphological features commonly used in distinguishing particular *Amazona* species.

Among the 30 known species of *Amazona* [2], *A. barbadensis* (the Yellow-shouldered Amazon) is the only species living exclusively in dry forests [5], inhabiting regions with xerophytic vegetation, frequenting desert shrublands dominated by cacti and low thorn-bushes or trees. Individuals of this species are found in several isolated populations in the coastal areas of Venezuela and across the Southern Caribbean region (the Venezuelan islands of Margarita and La Blanquilla as well as the Netherlands Antilles islands of Curacao and Bonaire) [6]. Its total population size is estimated at 2,500 to 10,000 individuals, and has been decreasing due to poaching, disappearance of natural habitat and the impact of non-native predators. The Yellow-shouldered Amazon is evaluated as ‘vulnerable’ by International Union for the Conservation of Nature and Natural Resources and is becoming a globally endangered parrot species. This has made the species the subject of many studies and conservation efforts.

The Yellow-shouldered Amazon is characterized by the yellow color of its shoulders, thighs, chin, upper part of the cheeks, area around the eyes, and over the ear openings (Figure S1). Forehead and lores are pale yellow or even white. The crown of this parrot sometimes has orange feathers. The lower part of cheeks and throat is usually bluish-green but sometimes also yellow. Although predominant yellow coloration of the shoulders is a characteristic feature of the species, some specimens with red patches on their shoulders were also observed.

In respect to plumage color, the Yellow-shouldered Amazon resembles most closely the Blue-fronted Amazon (*A. aestiva*). Individuals of *A. a. xanthopteryx* subspecies have also the yellow/red coloration of shoulders as well as yellow/white/blue coloration of the head. However, this triple coloration of the head is differentially distributed in these two species. Despite the plumage similarities, a close relationship between the two species has so far never been supported by molecular research. However, Rusello and Amato [7] found that both species are nested within the Yellow-Headed Amazon (YHA) complex.

The YHA complex has been described as ‘a taxonomic headache’ [8] because the number of recognized species varies according to different taxonomic classifications. Forshaw [3] considers a single species (*A. ochrocephala*) to exist, with eleven subspecies in the complex; whereas other authorities recognize three separate species (*A*. *ochrocephala*, *A*. *auropalliata*, *A*. *oratrix*) also with 11 subspecies [2], [4], [9].

According to the latter classification [2], the species *A. ochrocephala* (Yellow-crowned Amazon) includes three South American subspecies (*A*. *o*. *ochrocephala*, *A*. *o*. *xantholaema*, *A*. *o*. *nattereri*) and one Central American subspecies (*A. o. panamensis*). The species *A. auropalliata* (Yellow-naped Amazon) contains three Central American subspecies: *auropalliata*, *parvipes* and *caribaea*, whereas *A. oratrix* (Yellow-headed Amazon) is comprised of the other four Central American subspecies: *oratrix*, *tresmariae*, *belizensis* and *hondurensis*. The split of these taxa is based on characters such as extent and position of yellow plumage on head and thighs, plumage coloration at the bend of wings and lesser wing-coverts, bill and foot pigmentation and body size [3], [4]. Recently, the species *A. tresmariae* was distinguished from *A. oratrix* by the International Ornithological Committee (IOC) [10] based on results of molecular analyzes [11], as was proposed by Navarro-Sigüenza and Peterson [12]. In summary, according to the most recent classification [10], four different species of *Amazona* (*ochrocephala*, *auropallita*, *oratrix* and *tresmariae*) are currently recognized within the YHA complex.

Eberhard and Bermingham [11] presented a phylogenetic study of the YHA complex based on combined mitochondrial gene sequences with a total length of 2 515 bp (nd2, cox1, atp8, atp6). Besides ‘typical’ YHA species such as: *A. tresmariae*, *A. oratrix* (two subspecies), *A. auropalliata* and *A. ochrocephala* (one Central American and three South American subspecies), they also included the Blue-fronted Amazon (*A. aestiva*). Based on the four-gene data set, they found that the complex can be divided into three main lineages: the Central American (CA) lineage comprising all Central American taxa, the Northern South American (NSA) lineage represented by an individual *A. ochrocephala ochrocephala* from Colombia, and a South American (SA) lineage including *A. ochrocephala ochrocephala* (from Brazil), *A. ochrocephala nattereri*, *A. ochrocephala xantolaema* and *A. aestiva*. This surprising separation of the Colombian *A. o. ochrocephala* sample from the other South American taxa was further confirmed based on short cox1 sequence dataset (622 bp) including additional *A. o. ochrocephala* samples, one from Colombia and two from different localities in Venezuela [11]. Finally, the analysis of the cox1 sequences demonstrated a phylogenetic separation of Northern South American *A. o. ochrocephala* from Central South America taxa. These results suggested strong phylogeographic structure in the Central American lineage, which was lacking in the South American lineage.

Using only the short cox1 sequence, Eberhard and Bermingham [11] reported also a putative phylogenetic position for *A. barbadensis*. They showed that the species represents a distinct clade, which is sister to the whole YHA complex. However, their findings disagree with the phylogenetic position of *A. barbadensis* that was proposed by Rusello and Amato [7].

Rusello and Amato [7] presented a phylogenetic hypothesis for the genus *Amazona*, which was based on 3160 bp length concatenated alignment of mitochondrial (cox1, 12S, 16S) and nuclear (β-fibint 7, rp40, trop) loci. This apparently more detailed study of the YHA complex contained as many as nine among all eleven YHA subspecies and two subspecies of *A. aestiva*. However, it did not take into account the molecular data derived from *A. tresmariae* species. These results did not confirm the subdivision of the YHA complex into three species (*oratrix*, *auropalliata* and *ochrocephala*) but rather suggested its paraphyly. The authors found also that *A. aestiva* is nested within the YHA complex. On the other hand, in contrast to the results by Eberhard and Bermingham [11], Rusello and Amato [7] showed that the species *A. barbadensis* is nested within the YHA complex. Despite the apparent morphological similarities between the *A. barbadensis* and *A. aestiva xanthopteryx* subspecies, the former appeared to be a distinct lineage within the YHA complex, sister to *A. ochrocephala ochrocephala* as well as Central American (CA) and South American (SA) clades. This single *A. ochrocephala ochrocephala* sample, separated from the other South American taxa, seems to correspond to the Northern South American (NSA) lineage that was proposed by Eberhard and Bermingham [11]. However, it is only a supposition because analyses by Rusello and Amato [7] included no other *A. ochrocephala ochrocephala* individuals whereas the geographic origin of the analyzed sample was not given. The phylogenetic position of the *A. barbadensis* species remains also ambiguous because trees obtained by two methods showed different topologies [7]. One of them treats *A. barbadensis* as a distinct lineage whereas the second one suggests its phylogenetic relationship with *A. ochrocephala ochrocephala* (NSA lineage) with rather low bootstrap support.

Surprisingly, none of three main lineages (CA, SA, NSA) from the YHA complex was detected by Ottens-Wainright *et al*. [13]. Their analyses based on cytochrome *b* sequences showed the existence of closely related group including the following *Amazona* species: *A. aestiva*, *A. auropalliata*, *A. tresmariae*, *A. ochrocephala* (two genetically separated individuals), *A. barbadensis*, and two Lesser Antillean taxa, *A. arausiaca* and *A. versicolor*. The most basal position of this group was taken by *A. aestiva* whereas genetically diverse individuals of *A. ochrocephala* clustered together with the CA lineage taxa (*A. auropalliata*, *A. oratrix* and *A. tresmariae*). Moreover, their analyses showed that *A. barbadensis* is rather sister to both Lesser Antillean species than to the typical YHA complex.

So far, Ribas *et al*. [14] have presented the most informative study of the YHA complex including more samples with confirmed geographic origins. Their analysis was based on four concatenated mitochondrial gene sequences (1820 bp length): nd2, cox1, ATPase 6 and 8, which represented four *A. ochrocephala* subspecies, two *A. oratrix* subspecies, *A. auropalliata auropalliata* subspecies, *A. tresmariae* species and two *A. aestiva* subspecies. The results confirmed the existence of the three main lineages found previously by Eberhard and Bermingham [11]: the Central American (CA), South American (SA) and Northern South American (NSA) lineages. The SA lineage was further divided into two well-supported clades and occurred as a sister clade to the CA lineage. The CA lineage contained several strongly supported groups and resolved relationships between *auropalliata*, *oratrix* and *tresmariae*. Nevertheless, the results underlined the need for taxonomic revision of *Amazona ochrocephala* species. Four subspecies of *A. ochrocephala* (according to American Ornithologists’ Union [9]) did not create one monophyletic clade but were distributed to the three main lineages. Even sequences ascribed to the same subspecies were separated from each other. Six individuals of *A. ochrocephala ochrocephala* from Brazil were divided into two groups within the SA clade 2, whereas the Colombian individual of this subspecies was genetically divergent from others and represented the NSA lineage. Moreover, several *A. ochrocephala nattereri* specimens were scattered within SA lineage depending on their locality.

Unfortunately, *A. barbadensis* was not sampled by Ribas *et al*. [14] and its taxonomic position was not determined. Therefore, it is not still clear if *A. barbadensis* is closely related to the YHA complex [7], [11] or Lesser Antillean species [13]. Therefore, we used newly sequenced complete mitochondrial genome of *A. barbadensis* [15] to obtain more molecular markers useful in inferring the taxonomic position of this species. Using the most extensive data set with different combinations of markers and many phylogenetic approaches, we also tried to clarify relationships within the YHA complex and resolve several question concerning its phylogeography.

## Materials and Methods

### Phylogenetic Analyses

Phylogenetic analyses were performed on seven data sets (Table 1, Tables S1–S6) created from aligned sequences of mitochondrial genes: NADH dehydrogenase subunit 2 (nd2), cytochrome oxidase subunit I (cox1), ATPase 6 (atp6), ATPase 8 (atp8), cytochrome b (cytb), 12s rRNA (12s) and 16S rRNA (16s). Different combinations of the genetic markers, similar to those used by Ribas *et al*. [14] and Rusello and Amato [7], were applied. The original sets were modified by inclusion of additional sequences or exclusion of others to obtain alignments with larger number of sites. The sequences were aligned and initially analyzed in MEGA 5 [16].

**Table 1:** Characteristics of analysed data sets.

Data set	Number of sequences	Length of alignment [bp]
nd+cox+atp6+8^1^	63	1810
nd+cox^2^	35	1663
nd+cox+atp6+8^2^	31	2515
12s+16s+cox^3^	47	1485
12s+16s+cox+cytb^4^	31	2179
cox	69	513
cytb	35	694

1 based on Ribas et al. [14] data set;

2 based on Ribas et al. [14] and modified data set;

3 based on Rusello and Amato [7] and modified data set;

4 similar to the set^3^ but including also cytochrome *b*.

To infer phylogenetic trees, we applied four approaches: Bayesian analysis in MrBayes 3.2.1 [17] as well as maximum likelihood analyses in TreeFinder [18], PAUP* 4.0b [19] and morePhyML 1.14 [20] based on PhyML 3.0 [21].

In MrBayes analyses, we assumed separate mixed+I+G(5) models for each data partition (rRNA genes and three codon positions in each of protein-coding genes) to sample appropriate models across the substitution model space in the Bayesian MCMC analysis itself avoiding the need for a priori model testing [22]. In the analyses, two independent runs starting from random trees were applied, each using eight (for cox and 12s+16s+cox+cytb data sets) or four (for the rest sets) Markov chains. Trees were sampled every 100 generations for 10,000,000 generations. In the final analysis, we selected trees from the last 3,174,000 to 5,745,000 generations (depending on the data set) that reached the stationary phase and convergence (i.e. the standard deviation of split frequencies stabilized and was lower than the proposed threshold of 0.01).

In TreeFinder we applied search depth set to 2 and separate substitution models for each data partition (rRNA genes and three codon positions in each of protein-coding genes) as suggested by this program’s Propose Model module according to AIC, AICc, BIC and HQ criteria. Trees inferred with (more) PhyML and PAUP were based on the best-fit substitution models as found in jModeltest 2.1 [21], [23] among 1624 candidate models according to all four criteria: AIC, AICc, BIC and DT. The best heuristic search algorithm, nearest neighbor interchanges (NNI) and subtree pruning and regrafting (SPR), in (more)PhyML was applied whereas in PAUP, the final tree was searched from 10 starting trees obtained by stepwise and random sequence addition followed by the tree-bisection-reconnection (TBR) branch-swapping algorithm.

To assess support for particular clades, non-parametric bootstrap analyses were performed on 1000 replicates for TreeFinder and PAUP approaches. Additionally, we applied the Local Rearrangements–Expected Likelihood Weights (LR-ELW) method in TreeFinder and the approximate likelihood ratio test (aLRT) based on a Shimodaira-Hasegawa-like procedure in morePhyML [24].

Tree topologies assuming different relationships between the three main Yellow-headed *Amazona* clades (Central American, South American and North-South American clades) were compared according to approximately unbiased (AU), Shimodaira-Hasegawa (SH) and weighted Shimodaira-Hasegawa (wSH) tests, which were performed in Consel v0.20 [25] assuming 10,000,000 replicates. Site-wise log-likelihoods for the analyzed trees were calculated in TreeFinder under the best fitted substitution models. In these analyses, we considered both all sites and sites with different rate categories, which were created by successive elimination of sites with the highest substitution rate in the given data set.

Divergence times of Yellow-headed *Amazona* complex were estimated in TreeFinder using global rate minimum deformation (GRMD) and local rate minimum deformation (LRMD) methods for five data sets (nd+cox, nd+cox+atp6+8^2^, 12s+16s+cox, 12s+16s+cox+cytb and cytb) that included appropriate taxa for which divergence time was estimated by Schweizer *et al*. [26]. Based on their calculations, we used the following calibration points: *Pionus menstruus* – *A. dufresniana*/*A. aestiva*: 10.8 MYA, *A. dufresniana* – *A. pretrei*/*A. aestiva*: 6.4 MYA, *A. pretrei* – *A. aestiva*: 4.3 MYA.

## Results and Discussion

**Figure 1:**
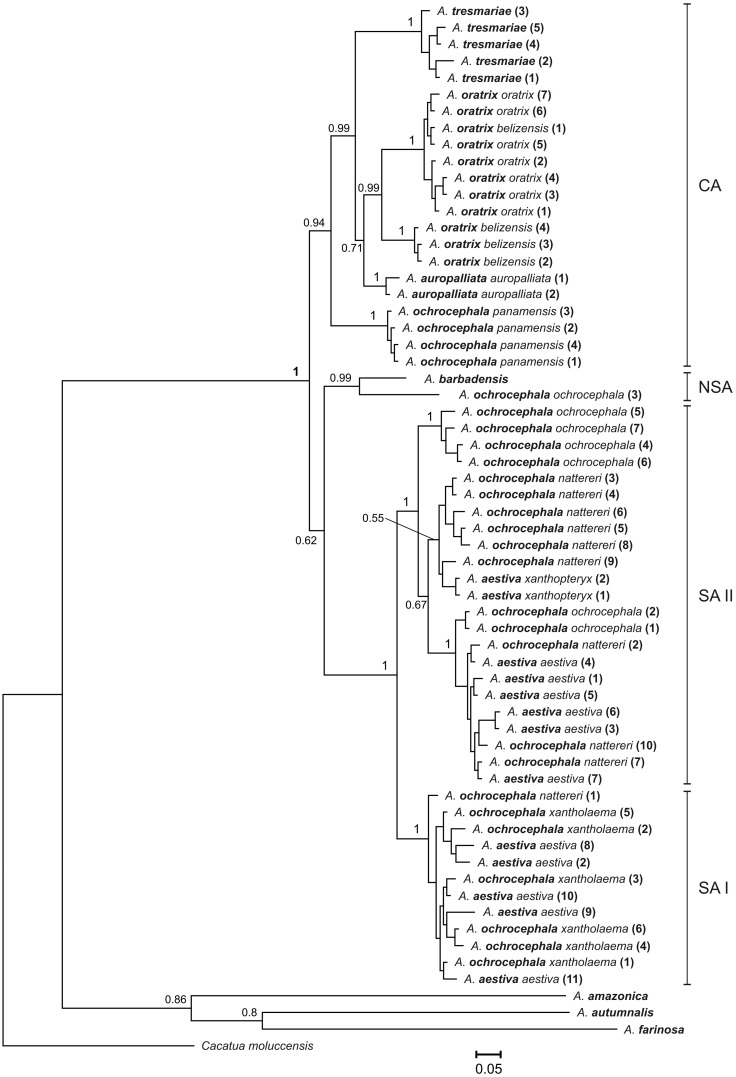
The Bayesian tree based on nd+cox+atp6+8^1^ data set. Values of posterior probabilities greater than 0.50 were shown on selected branches. Three main lineages of Yellow-Headed Amazons (YHA) are marked: Central American (CA), South American (SA) and Northern South American (NSA). Species name of analyzed *Amazona* (*A*.) individuals are marked in bold. Numbers at the end of some names correspond to the labeling of individuals listed in Table S1.

**Figure 2:**
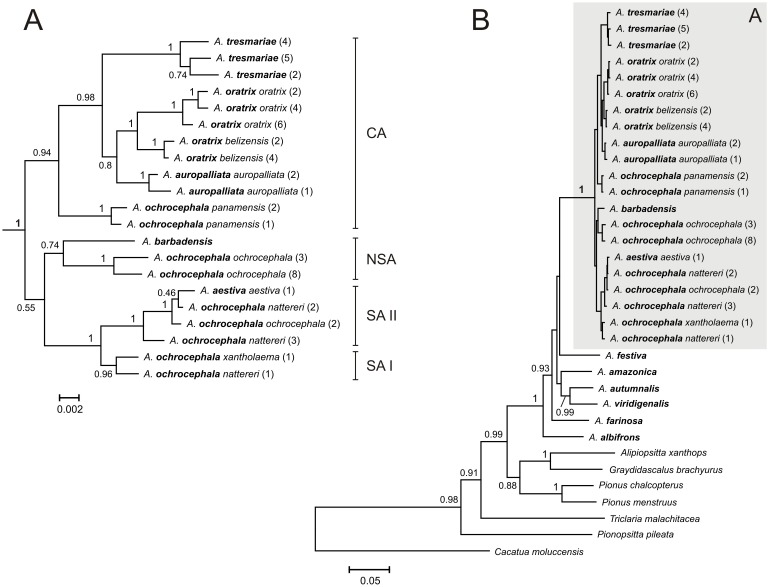
The Bayesian tree for the YHA complex (A) and all taxa (B) based on nd+cox^2^ data set. See Figure 1 for other explanations.

**Figure 3:**
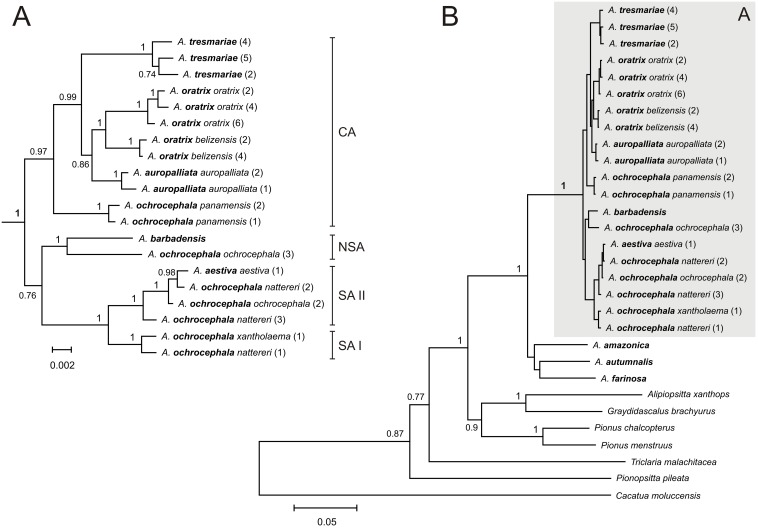
The Bayesian tree for the YHA complex (A) and all taxa (B) based on nd+cox+atp6+8^2^ data set. See Figure 1 for other explanations.

**Figure 4:**
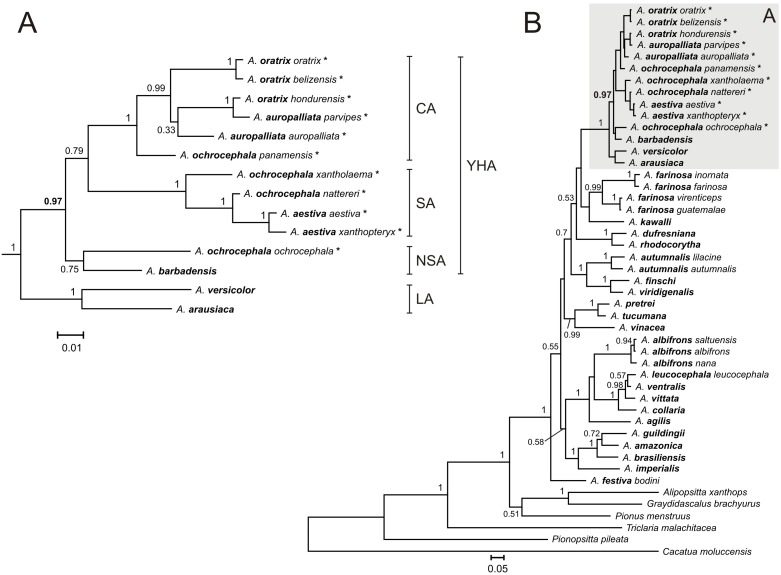
The Bayesian tree for the YHA complex and Lesser Antillean Amazons, LA (A) as well as all taxa (B) based on 12s+16s+cox^3^ data set. Asterisks at the end of some names indicate samples that were also used in further analysis based on the cytochrome oxidase data set (Figure 5). See Figure 1 for other explanations and Table S2 for more details.

**Figure 5:**
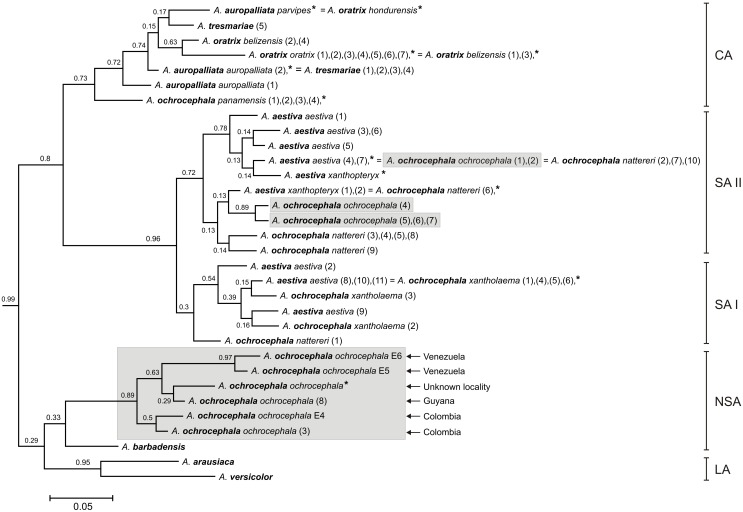
The Bayesian tree for the YHA complex and Lesser Antillean Amazons (LA) based on cytochrome oxidase data set. Individuals of *A. ochrocephala ochrocephala* subspecies are grey shadowed. Geographic locality (Venezuela, Colombia or Unknown) of Northern South American (NSA) individuals is noted. Numbers and asterisks at the end of names correspond to the labeling of individuals listed in Table S1 and Table S2 as well as used in previous analyzes based on nd+cox+atp6+8^1^ data set (numbers), nd+cox^2^ (numbers) and 12s+16s+cox^3^ data set (asterisks). See Figure 1 for other explanations and Table S3 for more details.

**Figure 6:**
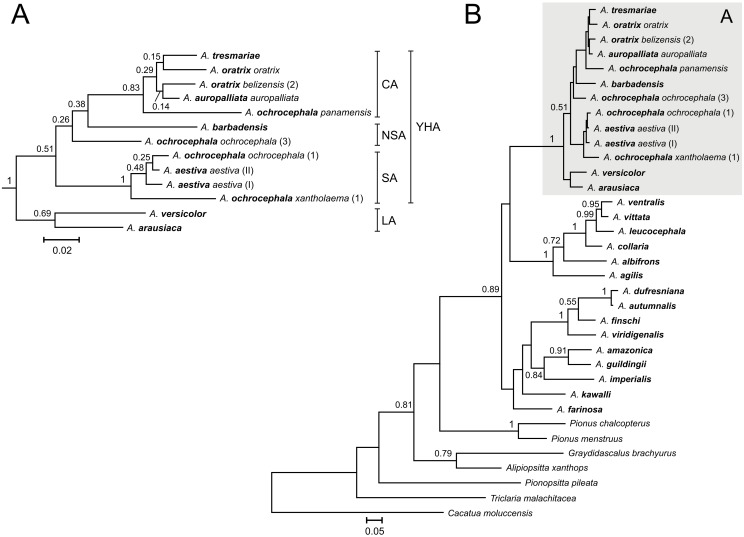
The Bayesian tree for YHA complex and Lesser Antillean Amazons, LA (A) as well as all taxa (B) based on cytochrome *b* data set. Numbers at the end of some names correspond to the labeling of individuals listed in Table S4. See Figure 1 for other explanations.

Phylogenetic analyses were carried out using four approaches on seven data sets. Generally, tree topologies (Figures 1–6, Figure S2) were similar to those obtained by other authors [7], [11], [14]. Yellow-Headed Amazons were most closely related to Lesser Antillean taxa, *A. arausiaca* and *A. versicolor*, and separated from other *Amazona* sequences.

### Phylogenetic Relationships of *A. barbadensis*

Newly obtained sequences from *A. barbadensis* enabled us to determine its relationship to other Yellow-Headed Amazons (YHA). All methods performed on five alignment sets consisted of at least two concatenated mitochondrial genetic markers unambiguously indicated close affiliation of *A. barbadensis* to *A. ochrocephala ochrocephala* that was represented by samples from Colombia, Guyana and Venezuela (Table 2, Figures 1–5, Figure S2). The support of this grouping was very high especially for two sets including NADH dehydrogenase, cytochrome oxidase subunit I, ATPase 6 and 8 (Figures 1 and 3). Moreover, the alignment containing rRNA and cytochrome oxidase sequences indicated lower support values of this grouping (Figure 4). Interestingly, addition of more sites from cytochrome *b* sequence to this set rather decreased these values (Figure S2). Phylogenies based on single markers, cytochrome oxidase subunit I (Figure 5) and cytochrome *b* (Figure 6) were too poorly resolved to give conclusive decision. Nevertheless, phylogenetic trees inferred in MrBayes and PAUP based on cytochrome oxidase alignment clustered *A. barbadensis* also with six *A. ochrocephala ochrocephala* sequences: two from Colombia, two from Venezuela and one from Guyana and one of unknown geographic locality (Table 2, Table S6, Figure 5). In the case of TreeFinder, this clade included also two Lesser Antillean taxa, *A. arausiaca* and *A. versicolor* but with low support (Table 2). The cluster of *A. barbadensis* and *A. ochrocephala ochrocephala* sequences correspond to the Northern South American (NSA) lineage, one of three forming the Yellow-Headed Amazon complex. The remaining two lineages, the Central American (CA) and the South American (SA) obtained also significant support in our studies.

**Table 2:** Values obtained for different methods and analysed data sets supporting Northern South American lineage including *A. barbadensis* and its relative *A. ochrocephala ochrocephala*.

Data set	MrBayes	LR-ELW	TF boot	PAUP boot	aLRT	PhyML boot
nd+cox+atp6+8^1^	0.99	94	92	73	0.93	77
nd+cox^2^	0.74	50	70	52	0.67	61
nd+cox+atp6+8^2^	1	88	92	86	0.90	88
12s+16s+cox^3^	0.75	57	63	50	0.76	59
12s+16s+cox+cytb^4^	0.38	43	48	41	0.70	46
cox	0.33	78*	40*	13	-	-

1 based on Ribas et al. [14] data set;

2 based on Ribas et al. [14] and modified data set;

3 based on Rusello and Amato [7] and modified data set;

4 similar to the set^3^ but including also cytochrome *b*;

*the clade contains also *A. arausiaca* and *A. versicolor*; LR-ELW - Local Rearrangements–Expected Likelihood Weights method; boot – bootstrap method; aLRT - the approximate likelihood ratio test based on a Shimodaira-Hasegawa-like procedure.

### Phylogenetic Relationships within the Central American Lineage

**Figure 7:**
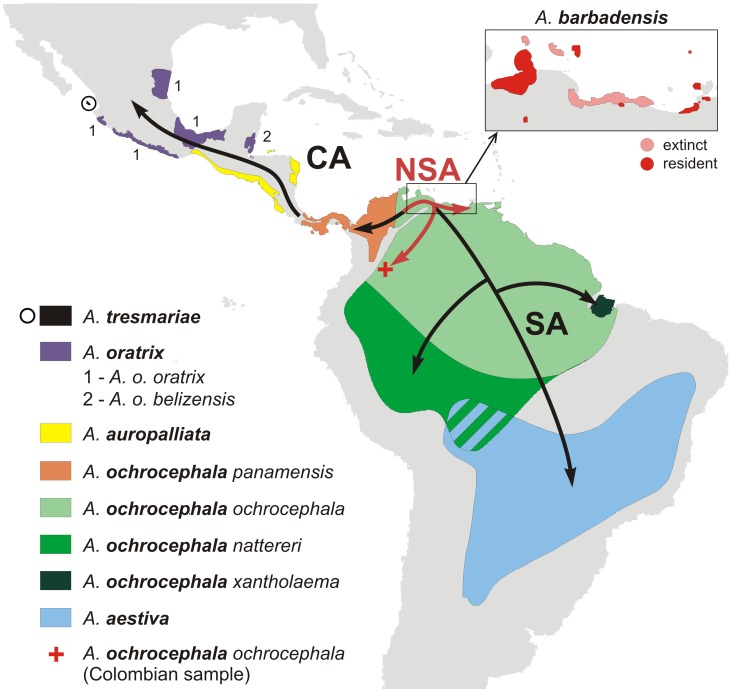
A possible scenario for YHA lineage evolution and their colonization of Central and South America. The most ancestral lineage is most likely NSA, whose distribution is restricted to the northernmost and north-west regions of South America. Their descendants colonized Central America through the Isthmus of Panama as well as southward regions of South America, giving rise to CA and SA lineages, respectively. The lineages diversified further into many taxa. However, more intense migrations and introgression likely did not lead to geographic differentiation of genetic markers in the SA lineage, in contrast to the CA lineage, which was subject to greater diversification by geographic locality.

*A. ochrocephala panamensis* was always placed at the base of the CA lineage (Figures 1–6, Figure S2), which suggests that an expansion of this lineage to Central America started from the territory of Panama and northwest Colombia after the rise of the Isthmus of Panama (Figure 7). Assuming the gradual diversification and expansion of *Amazona* species to subsequent regions located in the north and west, deep into Central America, one could expect that the subsequent branching of other CA taxa in phylogenetic trees corresponds to their successive north-westward geographic distribution.

However, the successive taxon diverging within the CA lineage was *A. tresmariae* found in Islas Marías (Mary Islands) located in the north and west limit of the CA lineage distribution, whereas the clade of *A. oratrix* and *A. auropalliata*, inhabiting more southward regions, split later (Figures 1–3). This clade obtained moderate support in our studies of three data sets: nd+cox+atp6+8^1^, nd+cox and nd+cox+atp6+8^2^ (MrBayes: 0.71, 0.80, 0.86; TreeFinder LR-ELW: 48, 54, 54; TreeFinder bootstrap: 45, 79, 84; PAUP bootstrap: 51, 78, 79; morePhyML: 0.74, 0.82, 0.78; PhyML bootstrap: 52, 83, 85, respectively). Trees for cytochrome oxidase (Figure 5) and cytochrome *b* (Figure 6) showed different topologies than described above but with very low support. The better resolved phylogenies suggest that the present localities of CA taxa may be different from the original ones and were colonized secondarily; or may be the remnants of a much wider distribution that has shrunk to the current one. It is possible that members of *A. tresmariae* lineage occurred originally in the south of Central America, whence they migrated northwards.

*A. oratrix* samples of the CA lineage clearly separated into two clades including subspecies *oratrix* and *belizensis*, respectively (Figures 1–3). Interestingly, one sequence from the *belizensis* subspecies was also present within the *A. oratrix oratrix* clade of the nd+cox+atp6+8^1^ tree (Figure 1). The clade had the maximal support in almost all applied methods. It is also noteworthy that *A. oratrix hondurensis* from the Sula Valley in Honduras [27] grouped significantly (MrBayes: 1; TreeFinder LR-ELW: 100; TreeFinder bootstrap: 96; PAUP bootstrap: 92; morePhyML: 0.98; PhyML bootstrap: 95) with *A. auropalliata parvipes* in the tree of 12s+16s+cox^3^ (Figure 4).

These results may indicate a misidentification of these samples or crossbreeding between *A. oratrix* subspecies as well as between *A. oratrix* and *A. auropalliata*. In agreement with that, cytochrome oxidase sequences appeared identical between these corresponding taxa (Figure 5). The same sequence showed also *A. auropalliata auropalliata* and *A. tresmariae*. Our data about *A. oratrix hondurensis* and *A. auropalliata parvipes* are compatible with findings of Lousada and Howell [28], who noted that *A. auropalliata caribaea* from the Bay Islands off the coast of Honduras shows plumage intermediate between that of the Atlantic slope *A. oratrix hondurensis* and the Pacific slope *A. auropalliata*. It was used as one of arguments to combine *A. auropalliata* and *A. oratrix* to one taxon [28]. In agreement with that, *A. oratrix* and *A. auropalliata* were always grouped together in the phylogenetic trees.

### Phylogenetic Relationships within the South American Lineage

The SA lineage clearly split into two significantly supported clades in our phylogenies (Figures 1–3, Figure 5) as in Ribas *et al*. [14]. Each of these two clades possessed a mixture of *Amazona* assigned to different species. Clade I included: *A. ochrocephala nattereri*, *A. ochrocephala xantholaema* and *A. aestiva aestiva*, whereas clade II included: *A. ochrocephala ochrocephala*, *A. ochrocephala nattereri*, *A. aestiva aestiva* and *A. aestiva xanthopteryx*. The separation of these clades corresponds neither to the course of the Amazon River nor Amazonian river dynamics in contrast to other taxa [29]–[31]. Their split may have resulted from other barriers, e.g. changes in type, composition and structure of vegetation that could occur during glacial cycles during the Pleistocene [32]–[37] and are not evident today [14]. The lack of monophyly for these species as well as the inconsistency between phylogenetic relationships and geographic distribution may result from misinterpretation of the morphological variation and hence wrong taxonomical classification especially in the case of unvouchered specimens [38], incomplete lineage sorting, and migrations accompanied with introgression [14]. In support of the latter, we identified identical cytochrome oxidase sequences assigned to the different species: *A. aestiva aestiva = A. ochrocephala ochrocephala = A. ochrocephala nattereri*; *A. aestiva xanthopteryx = A. ochrocephala nattereri* and *A. aestiva aestiva = A. ochrocephala xantholaema* (Figure 5). All these results, together with the clear separation of *A. ochrocephala* subspecies into the three main YHA lineages, indicate a need of revision of the whole *Amazona ochrocephala* taxon. There is some support for *A. ochrocephala panamensis* to be split from *A. ochrocephala* and form a separate species (*A. panamensis*), as it is the only representative of *A. ochrocephala* in the CA lineage. Moreover, in contrast to other *A. ochrocephala* subspecies, individuals of *A. o. panamensis* are characterized by smaller body size and pinkish-horn bill coloration [3], [4].

### Phylogenetic Relationships between Three Main Lineages of the YHA Complex

Although three lineages of the YHA complex were clearly recognized, the relationship between them was less obvious and depended on data sets rather than methods used (Table 3). Phylogenies based on nd+cox+atp6+8^1^, nd+cox and nd+cox+atp6+8^2^ data sets indicated the grouping of SA and NSA lineages for all methods with one exception (consensus from PhyML bootstrap trees). It suggested a closer relationship between the CA and NSA lineages than other possibilities. On the other hand, trees based on 12s+16s+cox, 12s+16s+cox+cytb and cox data sets suggested closer affiliation of SA and CA lineages by most methods. Only PhyML approaches using 12s+16s+cox+cytb and TreeFinder methods based on cytochrome oxidase preferred the CA and NSA clustering. In the latter case, the NSA lineage included also *A. arausiaca* and *A. versicolor*. Trees inferred on cytochrome *b* placed separated *A. barbadensis* and *A. ochrocephala ochrocephala*, representatives of the NSA lineage, and placed them basal to the CA lineage (Figure 6).

**Table 3:** Relationships between three main Yellow-headed *Amazona* lineages and their support values obtained for different methods and analysed data sets.

Data set	MrBayes	LR-ELW	TF boot	PAUP boot	aLRT	PhyML boot
nd+cox+atp6+8^1^	(S,NS)0.62	(S,NS)52	(S,NS)45	(S,NS)35	(S,NS)0.29	(S,NS)38
nd+cox^2^	(S,NS)0.55	(S,NS)49	(S,NS)54	(S,NS)46	(S,NS)0.33	(C,NS)42
nd+cox+atp6+8^2^	(S,NS)0.76	(S,NS)58	(S,NS)61	(S,NS)66	(S,NS)0.78	(S,NS)61
12s+16s+cox^3^	(S,C)0.79	(S,C)59	(S,C)65	(S,C)61	(S,C)0.79	(S,C)59
12s+16s+cox+cytb^4^	(S,C)0.54	(S,C)46	(S,C)47	(S,C)42	(C,NS)0.15	(C,NS)41
cox	(S,C)0.80	(C,NS*)98	(C,NS*)30	(S,C)33	(S,C)85	(S,C)38

1 based on Ribas et al. [14] data set;

2 based on Ribas et al. [14] and modified data set;

3 based on Rusello and Amato [7] and modified data set;

4 similar to the set^3^ but including also cytochrome *b*;

*the clade contains also *A. arausiaca* and *A. versicolor*; LR-ELW - Local Rearrangements–Expected Likelihood Weights method; boot – bootstrap method; aLRT - the approximate likelihood ratio test based on a Shimodaira-Hasegawa-like procedure.

C –Central American lineage, S – South American lineage, NS – Northern South American lineage including *A. barbadensis* and its relative *A. ochrocephala ochrocephala*.

However, in almost all approaches support values were low or at most moderate so it was difficult to favor one of these possibilities. Therefore we performed tests on tree topologies assuming different relationships between the three YHA lineages (Table 4 and 5). As would be expected, the comparison of the different scenarios (using all sites in the alignments) preferred none of them. However, when sites with the highest substitution rate in the given data set were successively eliminated, some topologies occurred with more statistically significant than others. Two topologies that clustered NSA with CA or SA lineages were favored, whereas the grouping of CA and SA lineages was significantly worse. The NSA+CA clade was preferred for nd+cox, 12s+16s+cox+cytb and cytb data sets, while the NSA+SA clade was chosen for 12s+16s+cox and two data sets including four markers, nd+cox+atp6+8^1^ and nd+cox+atp6+8^2^. Interestingly, the CA+SA clade obtained in trees based on all sites of 12s+16s+cox and 12s+16s+cox+cytb alignments was rejected when rapidly evolving sited were removed from these data sets. Moreover, the clade grouping two members of the NSA lineage, *A. barbadensis* and *A. ochrocephala ochrocephala*, was preferred for more conserved sites than for all position of cytochrome *b* alignment (Table 5). Although these results suggest that phylogenetic signal is too low to unambiguously select one of two scenarios (NSA + CA or NSA + SA), the close relationship of NSA to SA could be considered preferentially because was based on longer alignments both for all and conserved sites. This possibility is supported by the strong affiliation of *A. barbadensis* with the taxon *A. ochrocephala ochrocephala* whose members were also assigned to the SA lineage. The ambiguity in inferring relationships between YHA lineages was probably caused by their quick evolution and divergence in a short time frame, as is also indicated by very short internal branches leading to these lineages in phylogenetic trees. Incomplete lineage sorting and introgression between newly emerging clades before their final separation could also decrease resolution of the trees and obscure the deep relationships among taxa.

**Table 4:** Results of the approximately unbiased (AU), Shimodaira-Hasegawa (SH) and weighted Shimodaira-Hasegawa (wSH) tests for studied data sets and topologies assuming different relationships between three Yellow-headed *Amazona* lineages: C – Central American, S – South American, NS – Northern South American including *A. barbadensis* and its relative *A. ochrocephala ochrocephala*.

Data set	Selected sites	P-values from AU/SH/wSH tests for topology:		
		(S,NS)	(C,NS)	(S,C)
nd+cox+atp6+8^1^	all	0.775/0.763/0.755	0.333/0.284/0.450	0.212/0.293/0.482
	with rates <2	0.766/0.823/0.822	0.298/0.255/0.382	0.071/0.080/0.165
	with rates <1	1.000/1.000/1.000	**1e-04**/**2e-05**/**2e-05**	**3e-50**/**0**/**0**
	with rates <0.5	1.000/1.000/1.000	**1e-09**/**0**/**0**	**1e-07**/**0**/**0**
	with rates <0.1	1.000/1.000/1.000	**1e-07**/**0**/**0**	**6e-68**/**0**/**0**
nd+cox^2^	all	0.673/0.724/0.715	0.537/0.561/0.586	0.131/0.390/0.418
	with rates <2	0.959/0.897/0.898	0.064/0.113/0.197	0.065/0.112/0.206
	with rates <1	0.194/0.330/0.326	0.860/0.950/0.904	0.148/0.298/0.097
	with rates <0.5	**5e-04**/**0.046**/**3e-04**	0.940/1.000/0.999	0.067/0.116/0.081
	with rates <0.1	**0.003**/0.107/**0.006**	0.997/1.000/1.000	**6e-55**/**0**/**0**
nd+cox+atp6+8^2^	all	0.747/0.769/0.758	0.418/0.386/0.500	0.125/0.293/0.385
	with rates <2	0.996/0.999/0.998	**0.004**/**0.014/0.020**	**5e-08**/**1e-06**/**1e-06**
	with rates <1	0.918/0.939/0.934	0.095/0.097/0.166	**0.007**/**0.022**/**0.035**
	with rates <0.5	0.966/0.979/0.982	**0.034**/**0.040**/0.070	**5e-05**/**3e-04**/**5e-04**
	with rates <0.1	0.755/0.838/0.841	0.245/0.343/0.400	**1e-06**/**1e-04**/**7e-06**
12s+16s+cox^3^	all	0.235/0.230/0.414	0.239/0.230/0.422	0.826/0.775/0.775
	with rates <2	0.322/0.278/0.503	0.347/0.257/0.306	0.752/0.731/0.731
	with rates <1	0.757/0.952/0.859	0.110/0.467/0.153	0.330/0.347/0.349
	with rates <0.5	0.578/0.725/0.715	0.620/0.733/0.742	0.154/0.175/0.176
	with rates <0.1	0.896/0.935/0.972	0.192/0.483/0.288	**0.028**/**0.047**/**0.044**
12s+16s+cox+cytb^4^	all	0.147/0.394/0.451	0.515/0.514/0.565	0.670/0.722/0.712
	with rates <2	**0.037**/0.353/0.342	0.577/0.636/0.642	0.613/0.651/0.648
	with rates <1	0.127/0.180/0.146	0.835/0.922/0.922	0.263/0.356/0.330
	with rates <0.5	**0.002**/**0.001**/**0.001**	0.971/0.994/0.994	**0.038**/**0.067**/**0.061**
	with rates <0.1	**4e-05**/**0.001**/**0.001**	1.000/1.000/1.000	**7e-93**/**0**/**0**

1 based on Ribas et al. [14] data set;

2 based on Ribas et al. [14] and modified data set;

3 based on Rusello and Amato [7] and modified data set;

4 similar to the set^3^ but including also cytochrome *b*.

The tests were performed for all sites and sites with decreasing substitution rate. P-values of the best tree are underlined whereas those less than 0.05 are in bold.

**Table 5:** Results of the approximately unbiased (AU), Shimodaira-Hasegawa (SH) and weighted Shimodaira-Hasegawa (wSH) tests for the cytochrome *b* set and topologies assuming different relationships between three Yellow-headed *Amazona* lineages: C – Central American, S – South American, NS – Northern South American including *A. barbadensis* (Ab) and its relative *A. ochrocephala ochrocephala*.

Selected sites	P-values from AU/SH/wSH tests for topology:			
	(C,Ab)	(C,NS)	(S,NS)	(S,C)
all	0.771/0.816/0.852	0.572/0.713/0.652	0.211/0.272/0.484	0.220/0.262/0.462
with rates <2	0.450/0.636/0.531	0.867/0.897/0.943	0.116/0.236/0.417	0.104/0.234/0.402
with rates <1	0.887/0.894/0.938	0.200/0.621/0.392	**0.033**/0.091/0.197	**0.034**/0.082/0.175
with rates <0.5	0.096/0.519/0.061	0.937/0.996/0.998	**0.031**/0.178/0.300	**0.031**/0.103/**0.005**
with rates <0.1	**0.002**/0.187/**0.006**	0.998/1.000/1.000	**4E-92**/**4e-05**/**7e-06**	**1E-65**/**0**/**0**

The tests were performed for all sites and sites with decreasing substitution different site rate. P-values of the best tree are underlined whereas those less than 0.05 are in bold.

### Possible Scenario for the Evolution of YHA Taxa and their Colonization of the American Mainland

Based on these analyses, we can propose a scenario for the evolution of YHA taxa and their colonization of Central and South America (Figure 7). Since two Lesser Antillean Amazons, *A. arausiaca* and *A. versicolor*, are most closely related to YHA taxa and especially members of the NSA lineage (as found in some phylogenetic trees, Figure 5), they or their relatives seem to be good potential YHA ancestors. They probably colonized the northern coast of Venezuela, which also corresponds with the past and present distribution of *A. barbadensis* (Figure 7). In support of this, *A. barbadensis* lives or inhabited in the past also some islands in the Lesser Antilles. Then the NSA taxa represent likely descendants of the YHA ancestors. After the colonization of the mainland, rapid evolution and divergence into new lineages accompanied the occupation of new habitats and/or free ecological niches. According to our estimates (Table 6), the migration to the mainland and emergence of the YHA complex happened about million years ago and could be related to climatic changes due to glacial cycles [39], [40].

**Table 6:** Divergence time in million years estimated by two methods for analysed data sets.

Data set	GRMD	LRMD
nd+cox^2^	0.94	0.99
nd+cox+atp6+8^2^	1.04	0.99
12s+16s+cox^3^	1.22	1.16
12s+16s+cox+cytb^4^	1.24	1.23
cytb	1.13	1.11

2 based on Ribas et al. [14] and modified data set;

3 based on Rusello and Amato [7] and modified data set;

4 similar to the set^3^ but including also cytochrome *b*.

A further colonization event to Central America through the already existing Isthmus of Panama [41] initiated the origin of the CA lineage (Figure 7). This is in agreement with the distribution of *A. ochrocephala panamensis*, which occupies geographic regions adjacent to those of the NSA taxa and was placed at the base of the CA lineage in phylogenetic trees (Figures 1–6, Figure S2). The southward expansion to the South America proceeded somewhat simultaneously. However, greater gene flow or migration may have occurred between NSA and SA than between more isolated CA taxa, as is suggested by tree topology tests, in which the NSA+SA clade was preferred by larger data sets (Table 4). The migrations and introgression could prevent geographic differentiation of genetic markers in the SA lineage, despite the larger occupied area, in contrast to the CA lineage [14]. It seems that changes during the Late Pleistocene in the Amazon Basin and adjacent regions inhabited by SA taxa were insufficient to cause distinct differentiation of species corresponding to their geographic distribution. Forests only in parts of eastern lowland Amazonia were at times more open and savannas may have developed only locally as a result of drier or more seasonal conditions. However, the majority of the Amazon rainforest remained intact [37], [42], [43]. In contrast to that, the CA taxa could undergo more intense diversification correlated with their geographic locality because of stronger influence of climatic changes on their habitat alterations and fragmentations related with glacial cycles. In fact it has been shown that high elevated regions of Central America were glaciated during the Late Pleistocene [44] with cooler conditions influencing also lowland forest vegetation and moisture pattern throughout the Central America [45]–[49]. This hypothesis corresponds with the results of Weir [50] who found higher speciation rates in the highland than lowland Neotropical birds during the last one million years because of climatic and glacial fluctuations. In agreement with this, CA taxa are mainly highland and upland species, whereas SA taxa favor lowlands. Nevertheless, further studies involving more samples from the whole distribution of YHA taxa represented by more genetic markers, including completely sequenced mitochondrial genomes, are necessary to verify the suggested scenario.

## Supporting Information

Figure S1 Individual of Yellow-shouldered Amazon (*Amazona barbadensis*) whose sequences were analysed in the paper.
(TIF)

Figure S2 The Bayesian tree for YHA complex and Lesser Antillean Amazons, LA (A) as well as all taxa (B) based on 12s+16s+cox+cytb^4^ data set. See Figure 1 for other explanations and Table S5 for more details.
(TIF)

Table S1 List of taxa, individuals and sequences included in: nd+cox+atp6+8^1^, nd+cox^2^ and nd+cox+atp6+8^2^ data sets. # - number of the species individual. Length - the length of the whole combined mitochondrial sequence.
(XLSX)

Table S2 List of taxa and sequences included in the 12s+16s+cox^3^ data set. Length - the length of the whole combined mitochondrial sequence.
(XLSX)

Table S3 List of taxa, individuals and sequences included in the cytochrome oxidase data set.
(XLSX)

Table S4 List of taxa, individuals and sequences included in the cytochrome *b* data set.
(XLSX)

Table S5 List of taxa and sequences included in the 12s+16s+cox+cytb^4^ data set. Length - the length of the whole combined mitochondrial sequence.
(XLSX)

Table S6 List of studied specimens including their voucher number, geographic location, locality code, references and institution hosted them.
(XLSX)

## References

[1] Schodde R, Remsen JV, Schirtzinger EE (2013) Higher classification of New World parrots (Psittaciformes; Arinae), with diagnoses of tribes. Zootaxa 3691(5): 591–596. 26167605 10.11646/zootaxa.3691.5.5

[2] Clements JF, Schulenberg TS, Iliff MJ, Sullivan BL, Wood CL, et al. (2013) The eBird/Clements checklist of birds of the world: Version 6.8. Available: http://www.birds.cornell.edu/clementschecklist/download. Accessed 2014 02.

[3] Forshaw JM (2010) Parrots of the World. London: A & C Black Publishers Ltd. 328 p.

[4] Juniper T, Parr M (1998) Parrots: a Guide to Parrots of the World. New Haven, London: Yale University Press. 584 p.

[5] Briceño-Linares JM, Rodríguez JP, Rodríguez-Clarkb KM, Rojas-Suárez F, Millán PA, et al (2011) Adapting to changing poaching intensity of Yellow-shouldered parrot (*Amazona barbadensis*) nestlings in Margarita island, Venezuela. Biol Cons. 4: 1188–1193.

[6] BirdLife International (2012) Aratinga brevipes. In: IUCN 2012. IUCN Red List of Threatened Species. Version 2012.2. <www.iucnredlist.org>. Accessed 2013 May 02.

[7] Russello MA, Amato G (2004) A molecular phylogeny of Amazona: implications for Neotropical parrot biogeography, taxonomy, and conservation. *Mol. Phylogenet.* Evol. 30: 421–437. 10.1016/s1055-7903(03)00192-1 14715233

[8] Howell SNG, Webb S (1995) A Guide to the Birds of Mexico and Northern Central America. Oxford: Oxford University Press. 1010 p.

[9] American Ornithologists Union (2013) Checklist of North American Birds, 7th ed. American Ornithologists’ Union. Washington, D.C.

[10] Gill F, Donsker D (2013) IOC World Bird List (v 3.4). Available: http://www.worldbirdnames.org Accessed 2013 11.

[11] Eberhard JR, Bermingham E (2004) Phylogeny and biogeography of the Amazona ochrocephala (Aves: Psittacidae) complex. Auk 121: 318–332.

[12] Navarro-SigüenzaI AG, Townsend Peterson A (2004) An alternative species taxonomy of the birds of Mexico. Biota Neotrop. 4: 2.

[13] Ottens-Wainright P, Kenneth MH, Eberhard JR, Burke RI, Wiley JW, et al (2004) Independent geographic origin of the genus *Amazona* in the West Indies. Journal of Caribbean Ornithology 17: 23–49.

[14] Ribas CC, Tavares ES, Yoshihara C, Miyaki CY (2007) Phylogeny and biogeography of yellow-headed and blue-fronted parrots (*Amazona ochrocephala* and *Amazona aestiva*) with special reference to the South American taxa. Ibis 149: 564–574.

[15] Urantowka AD, Hajduk K, Kosowska B (2013) Complete mitochondrial genome of endangered Yellow-shouldered Amazon (*Amazona barbadensis*): two control region copies in parrot species of the Amazona genus. Mitochondrial DNA 24(4): 411–3. 23406580 10.3109/19401736.2013.766177

[16] Tamura K, Peterson D, Peterson N, Stecher G, Nei M, et al (2011) MEGA5: Molecular Evolutionary Genetics Analysis using Maximum Likelihood, Evolutionary Distance, and Maximum Parsimony Methods. Molecular Biology and Evolution 28: 2731–2739. 21546353 10.1093/molbev/msr121 PMC3203626

[17] Ronquist F, Teslenko M, van der Mark P, Ayres DL, Darling A, et al (2012) MrBayes 3.2: efficient Bayesian phylogenetic inference and model choice across a large model space. Systematic Biology 61: 539–542. 22357727 10.1093/sysbio/sys029 PMC3329765

[18] Jobb G, von Haeseler A, Strimmer K (2004) TREEFINDER: a powerful graphical analysis environment for molecular phylogenetics. BMC Evolutionary Biology 4: 18. 15222900 10.1186/1471-2148-4-18 PMC459214

[19] Swofford DL (1998) PAUP*. Phylogenetic analysis using parsimony (*and other methods). Version 4. Sunderland, MA: Sinauer Associates.

[20] Criscuolo A (2011) morePhyML: improving the phylogenetic tree space exploration with PhyML 3. Mol Phylogenet Evol 61: 944–948. 21925283 10.1016/j.ympev.2011.08.029

[21] Guindon S, Dufayard JF, Lefort V, Anisimova M, Hordijk W, et al (2010) New Algorithms and Methods to Estimate Maximum-Likelihood Phylogenies: Assessing the Performance of PhyML 3.0. Systematic Biology 59(3): 307–21. 20525638 10.1093/sysbio/syq010

[22] Huelsenbeck JP, Larget B, Alfaro ME (2004) Bayesian phylogenetic model selection using reversible jump Markov chain Monte Carlo. Molecular Biology and Evolution 21: 1123–1133. 15034130 10.1093/molbev/msh123

[23] Darriba D, Taboada GL, Doallo R, Posada D (2012) jModelTest 2: more models, new heuristics and parallel computing. Nature Methods 9: 772. 10.1038/nmeth.2109 PMC4594756 22847109

[24] Anisimova M, Gascuel O (2006) Approximate likelihood-ratio test for branches: a fast, accurate, and powerful alternative. Syst Biol. 55: 539–52. 10.1080/10635150600755453 16785212

[25] Shimodaira H, Hasegawa M (2001) CONSEL: for assessing the confidence of phylogenetic tree selection. Bioinformatics 17: 1246–7. 11751242 10.1093/bioinformatics/17.12.1246

[26] Schweizer M, Seehausen O, Hertwig ST (2011) Macroevolutionary patterns in the diversification of parrots: effects of climate change, geological events and key innovations. J. Biogeogr. 38: 2176–2194.

[27] Howell SNG, Webb S (1992) New and noteworthy bird records from Guatemala and Honduras. *Bull. Brit. Orn.* . Club 112: 42–49.

[28] Lousada SA, Howell SNG (1996) Distribution, variation and conservation of yellow-headed parrots in northern Central America. Cotinga 5: 46–53.

[29] Ayres JMC, Clutton-Brock TH (1992) River boundaries and species range size in Amazonian primates. *Am.* Nat. 140: 531–537. 10.1086/285427 19426056

[30] Ribas CC, Aleixo A, Nogueira AC, Miyaki CY, Cracraft J (2012) A palaeobiogeographic model for biotic diversification within Amazonia over the past three million years. Proc Biol Sci. 279(1729): 9–681. 10.1098/rspb.2011.1120 PMC3248724 21795268

[31] Lutz HL, Weckstein JD, Patané JS, Bates JM, Aleixo A (2013) Biogeography and spatio-temporal diversification of Selenidera and Andigena Toucans (Aves: Ramphastidae). Mol Phylogenet Evol. 69(3): 873–83. 10.1016/j.ympev.2013.06.017 23831458

[32] Haffer J (1969) Speciation in Amazonian forest birds. Science 165: 131–137. 17834730 10.1126/science.165.3889.131

[33] Haffer J, Prance GT (2001) Climate forcing of evolution in Amazonia during the Cenozoic: on the refuge theory of biotic differentiation. Amazoniana 16: 579–607.

[34] Bush MB (1994) Amazonian speciation: a necessarily complex model. J. Biogeogr. 21: 5–17.

[35] Cowling SA, Maslin MA, Sykes MT (2001) Paleovegetation simulations of lowland Amazonia and implications for neotropical allopatry and speciation. Quaternary Research 55: 9–140.

[36] Cowling SA (2004) Tropical forest structure: a missing dimension to Pleistocene landscapes. Journal of Quaternary Science 19: 43–733.

[37] Maslin M, Mahli Y, Phillips O, Cowling S (2005) New views on an old forest: assessing the longevity, resilience and future of the Amazon rainforest. T I Brit Geogr NS 30: 477–499.

[38] Peterson AT, Moyle RG, Nyári AS, Robbins MB, Brumfield RT, et al (2007) The need for proper vouchering in phylogenetic studies of birds. Mol Phylogenet Evol. 45(3): 1042–1044. 10.1016/j.ympev.2007.08.019 17962047

[39] Braun DD (2004) The Glaciation of Pennsylvania USA. In: Ehlers J, Gibbard PL, editors. Quaternary Glaciations - Extent and Chronology: Part II: North America. Elsevier 237–242.

[40] Cohen KM, Gibbard P (2011) Global chronostratigraphical correlation table for the last 2.7 million years. Subcommission on Quaternary Stratigraphy. Cambridge, England: International Commission on Stratigraphy. Accessed 2013 11.

[41] Coates AG (1997) The forging of Central America. In: Coates AG, editor. Central America: a natural history. New Haven: Yale University Press. 1–37.

[42] Colinvaux PA, Oliveira PE (2000) Palaeoecology and climate of the Amazon basin during the last glacial cycle. J Quaternary Sci. 15: 347–356.

[43] Behling H, Bush M, Hooghiemstra H (2010) Biotic development of Quaternary Amazonia: a palynological perspective. In: Hoorn C, Wesselingh FP, editors. Amazonia, Landscape and Species Evolution: A Look into the Past. Blackwell Publishing. 335–345.

[44] Lachniet MS (2004) Late Quaternary Glaciation of Costa Rica and Guatemala Central America. In: Ehlers J, Gibbard PL, editors. Quaternary Glaciations - Extent and Chronology: Part III: South America, Asia, Africa, Australia, Antarctica. Elsevier 135–138.

[45] Bush MB, Colinvaux PA (1990) A pollen record of a complete glacial cycle from lowland Panama. Journal of Vegetation Science 1: 105–118.

[46] Hooghiemstra H, Cleef AM, Noldus CW, Kappelle M (1992) Upper Quaternary vegetation dynamics and palaeoclimatology of the La Chonta bog area (Cordillera de Talamanca, Costa Rica). Journal of Quaternary Science 7: 205–225.

[47] Islebe GA, Hooghiemstra H (1997) Vegetation and climate history of montane Costa Rica since the last glacial. Quaternary Science Reviews 16: 589–604.

[48] Horn SP, Sanford RL, Dilcher D, Lott TA, Renne PR, et al (2003) Pleistocene plant fossils in and near La Selva Biological Station, Costa Rica. Biotropica 35: 434–441.

[49] Bush MB, Correa-Metrio AY, Hodell DA, Brenner M, Anselmetti F S, et al. (2009) Re-evaluation of Climate Change in Lowland Central America During the Last Glacial Maximum Using New Sediment Cores from Lake Petén Itzá, Guatemala. In: Vimeux F, et al.. editors. Past Climate Variability in South America and Surrounding Regions: Developments in Paleoenvironmental Research Volume 14. Springer Science+Business Media BV. 113–128.

[50] Weir JT (2006) Divergent timing and patterns of species accumulation in lowland and highland Neotropical birds. Evolution 60: 842–855. 16739464

