# Supplementary figures and images for: Phylogeny of Amazona barbadensis and the Yellow-Headed Amazon Complex (Aves: Psittacidae): A New Look at South American Parrot Evolution
Source: PLoS One. 2014 May 13;9(5):e97228. doi: 10.1371/journal.pone.0097228 (PMC4019560; doi:10.1371/journal.pone.0097228)

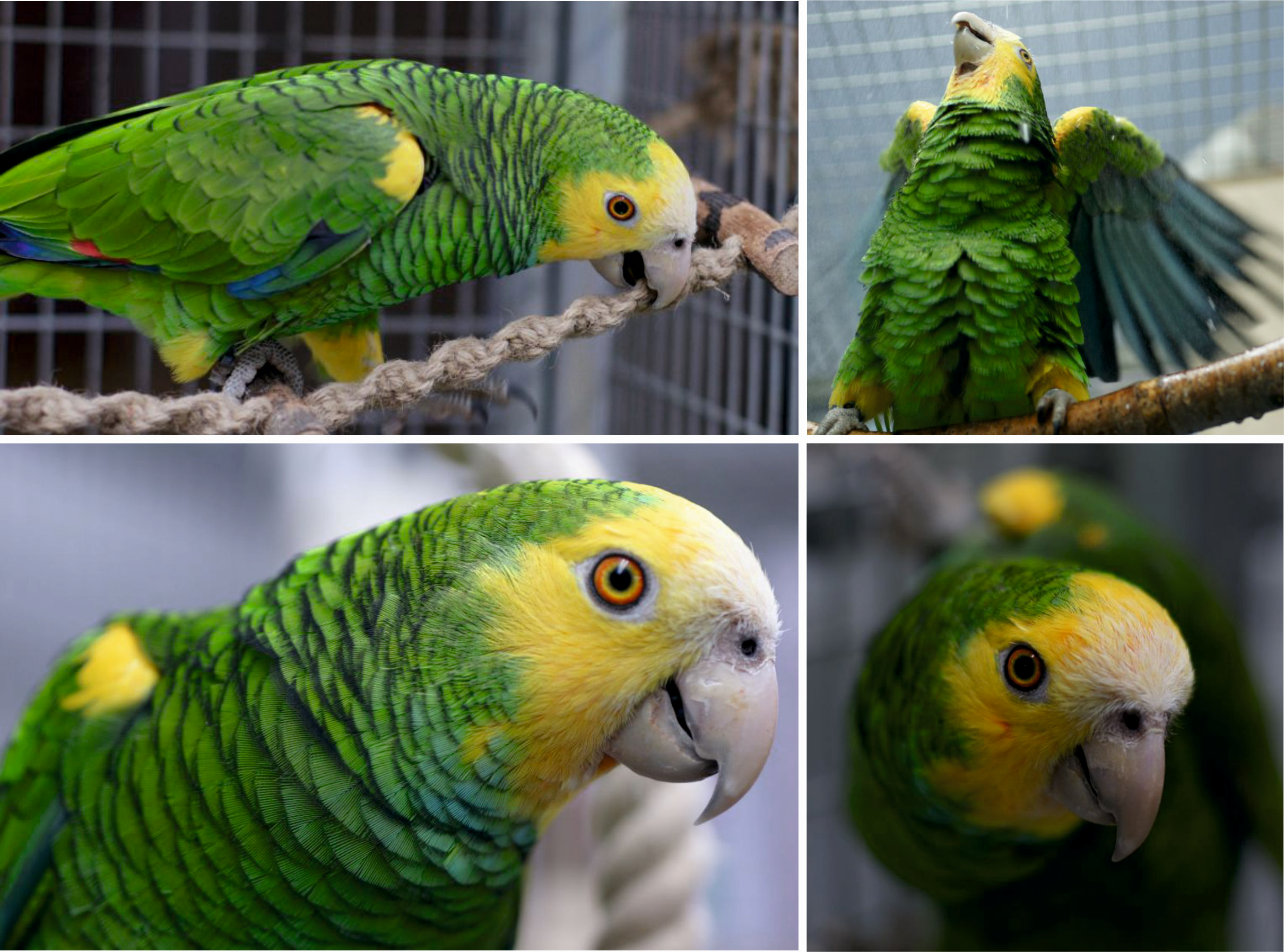

Supplement: Figure S1 — Individual of Yellow-shouldered Amazon (Amazona barbadensis) whose sequences were analysed in the paper. (TIF) [file pone.0097228.s001.tif]

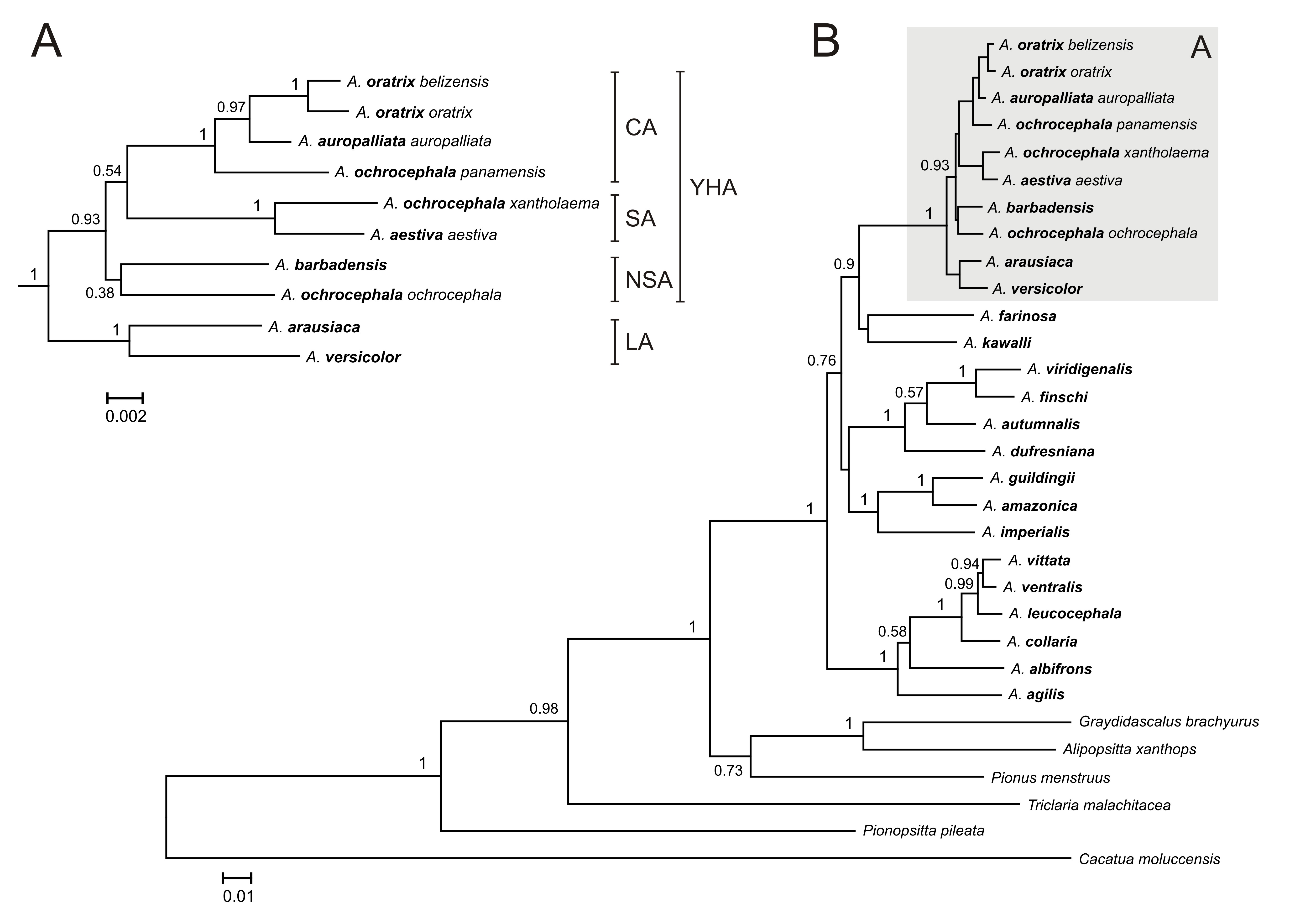

Supplement: Figure S2 — The Bayesian tree for YHA complex and Lesser Antillean Amazons, LA (A) as well as all taxa (B) based on 12s+16s+cox+cytb4 data set. See Figure 1 for other explanations and Table S5 for more details. (TIF) [file pone.0097228.s002.tif]
